# Factors Associated with Chest Tube Placement in Blunt Trauma Patients with an Occult Pneumothorax

**DOI:** 10.1155/2019/9274697

**Published:** 2019-09-02

**Authors:** Michael Paplawski, Swapna Munnangi, Jody C. Digiacomo, Edwin Gonzalez, Ashley Modica, Shawndeep S. Tung, Catherine Ko

**Affiliations:** ^1^Department of Surgery, Nassau University Medical Center, East Meadow, NY, USA; ^2^Department of Plastic Surgery, University of South Florida Morsani School of Medicine, Tampa, FL, USA; ^3^Banner MD Anderson Cancer Center, Sun City West, AZ, USA; ^4^Doctors Center, Bridgeport, CT, USA

## Abstract

**Background:**

An occult pneumothorax is identified by computed tomography but not visualized by a plain film chest X-ray. The optimal management remains unclear.

**Methods:**

A retrospective review of an urban level I trauma center's trauma registry was conducted to identify patients with occult pneumothorax over a 2-year period. Factors predictive of chest tube placement were identified using univariate and multivariate logistic regression analysis.

**Results:**

A total of 131 patients were identified, of whom 100 were managed expectantly with an initial period of observation. Ultimately, 42 (32.0%) patients received chest tubes and 89 did not. The patients who received chest tubes had larger pneumothoraces at initial assessment, a higher incidence of rib fractures, and an increased average number of rib fractures, of which significantly more were displaced.

**Conclusions:**

Displaced rib fractures and moderate-sized pneumothoraces are significant factors associated with chest tube placement in a victim of blunt trauma with occult pneumothorax. The optimal timing for the first follow-up chest X-ray remains unclear.

## 1. Introduction

Occult pneumothorax (OPTX) is diagnosed when a collection of air within the pleural cavity is found on computerized tomography (CT) scan, but not detected on anterior-posterior chest radiography (CXR). With the significant utilization of CT scans in the evaluation of blunt force trauma patients, the incidence of OPTX is rising [1, 2]. The incidental discovery of an OPTX in a blunt trauma patient is estimated to be 2–10% [3, 4]. Incidental findings on thoracic CT that may be otherwise clinically insignificant are associated with increased costs, longer observation times in the emergency department (ED), and slower patient throughput [5, 6]. With thoracic injuries being a significant cause of morbidity and mortality in trauma patients, the superior sensitivity of thoracic CT scans over CXRs will remain an essential component of the initial evaluation of a trauma patient [7]. When an OPTX is identified by a CT scan done, the clinician must ask “Is this OPTX merely an incidental finding with no clinical relevance, or will it potentially require an intervention? In the days before CT, this was the same challenge for stab wounds to chest. Those which developed subsequent to an initial normal CXR were labelled “delayed pneumothorax” [8, 9].

Tube thoracostomy is not a benign procedure, and it is therefore important to anticipate which patients will be more likely to require a chest tube for the management of their OPTX. There is limited data available that provides a clinician with predictive criteria to aid in the management of an OPTX in blunt trauma patients. Observation of most of the patients with an OPTX is generally considered a safe option [10]. What is not clear is which patient characteristics are associated with failure of observation and make them more likely to require a chest tube during their hospitalization [11–13]. In an effort to assist the clinical decision-making process, our study aims to evaluate the management of blunt trauma patients with an OPTX and to identify the factors associated with the need for chest tube placement in these patients.

## 2. Methods

Nassau University Medical Center (NUMC) is a 502 bed, level I trauma center located in Nassau County, New York. NUMC is a tertiary care and safety net institution for the region and admits 1500 to 1700 trauma patients per year. Using the NUMC trauma registry, 131 patients identified with a diagnosis of occult pneumothorax demonstrated on CT but not on chest X-ray between January 2012 and December 2013 were included in this study. The hospital trauma registry was used to evaluate all trauma patients with pneumothoraces during the above-noted timeframe, and records were individually evaluated to determine which patients had occult pneumothoraces. The patients were considered to have an occult pneumothorax secondary to trauma if their initial trauma chest X-ray did not demonstrate a pneumothorax, but their initial trauma CT scan did demonstrate a pneumothorax [14–16]. All patients with penetrating trauma and those with incomplete medical records were excluded from the study sample. All of the inpatient admissions, radiology reports, and outpatient clinic records related to the traumatic injuries were reviewed. Patient demographic data and variables such as prehospital and admission vitals, mechanism of injury, Injury Severity Score (ISS), probability of survival (POS), Revised Trauma Score (RTS), Glasgow Coma Scale (GCS), intubation status, hospital and intensive care unit (ICU), length of stay (LOS), in-hospital complications, and disposition were collected from the trauma registry. Number and type of rib fractures, size of pneumothorax, and number of chest X-rays performed were examined from the medical record of the patients. Rib fractures were determined by attending radiologist evaluation and were considered displaced if the bony cortex of the rib was malaligned. Mortality in the study represents in-hospital mortality defined as death occurring during the course of hospital stay. The study protocol was approved by NUMC's Institutional Review Board.

### 2.1. Statistical Analysis

Descriptive statistics were performed on all variables in this study. Categorical variables were described as frequency distributions and percentages. Continuous variables were calculated as means ± standard deviations. To examine differences between groups, Pearson's chi-square test or Fisher's exact test was used for categorical variables, while Student's *t*-test or Mann-Whitney *U* test was used for continuous variables. Logistic regression analysis adjusting for confounders was performed to identify significant factors associated with chest tube placement, the primary outcome of interest. Confounders were chosen from the variables that were found to be independently associated with the primary outcome of interest. A *p* value of less than 0.05 was considered to be statistically significant. SAS version 9.4 (SAS Institute, Cary, NC) was used as the statistical tool in this study.

## 3. Results

During the two-year study period (2012-2013), 2402 patients were admitted to the trauma center. A total of 131 patients were found to have an OPTX after blunt trauma, of whom 42 (32.06%) patients ultimately received chest tubes and 89 (67.94%) did not. Baseline characteristics of the study population with the subgroups of occult pneumothorax patients that received a chest tube and those that did not are presented in Table 1.

Of the 131 patients determined to have an OPTX, 100 were managed expectantly with an initial period of observation while 31 received chest tubes. This was determined by the attending trauma surgeon based on factors such as hemodynamic instability, positive pressure ventilation, need for a surgical procedure, or other clinical factors.

Of the 100 patients given an initial period of observation, the first follow-up CXR showed no progression of the OPTX for 94 patients. Three of the patients received chest tubes based on the clinical determination of the attending trauma surgeon. A second follow-up CXR was obtained in 62 patients, which showed no progression of the OPTX in 61. Progression of the OPTX was noted in two patients, of whom one received a chest tube and one did not.

Of the four patients given an initial period of observation whose first follow-up CXR showed progression of the OPTX, one received a chest tube. The other three patients received a second follow-up CXR, which demonstrated no further progression in two patients. One patient had further progression of the OPTX and received a chest tube.

No significant differences were observed in age, gender, smoking status, and mechanism of blunt injury between the two sample groups who ultimately received chest tubes and those who did not. As compared to patients who did not receive a chest tube, patients with an OPTX who did receive a chest tube had a statistically significant higher incidence of rib fractures (90.48% vs. 66.29%; *p*=0.0032), an increased average number of ribs fractured (5.98 ± 4.21 vs. 3.55 ± 3.95; *p*=0.0017), of which significantly more were displaced (71.05% vs. 44.07%; *p*=0.0056). OPTX patients that received a chest tube also had a higher ISS (25.76 ± 14.15 vs. 18.74 ± 8.23; *p*=0.0042), higher RTS (6.76 ± 1.61 vs. 7.59 ± 0.76; *p* ≤ 0.0031), and lower POS (0.80 ± 0.23 vs. 0.93 ± 0.14; *p*=0.0020). They required intubation more often (75.61% vs. 17.98%; *p* ≤ 0.0001), had longer hospital LOS (18.29 ± 17.88 vs. 5.63 ± 6.59 days; *p* ≤ 0.0001) and ICU LOS (6.64 ± 6.85 vs. 1.89 ± 3.00 days; *p* ≤ 0.0001), and had more CXRs performed (12.69 ± 9.41 vs. 3.34 ± 2.88; *p* ≤ 0.0001). They also has a higher in-hospital mortality rate (16.67% vs. 5.62%; *p*=0.0408) and higher in-hospital complication rate (35.71% vs. 3.37%; *p*=0.0003) (Table 1).

The average time to chest tube placement was 16.43 ± 32.07 hours. The majority (78.57%) of the patients that received a chest tube did so within 24 hours of admission.

In the multivariable regression model, factors that were found to be significantly associated with chest tube placement in OPTX patients included displaced rib fractures (OR = 4.166, 95% CI 1.455–11.930; *p*=0.0078), intubation (OR = 15.581, 95% CI 4.676–51.914; *p* < 0.0001), and moderate-sized occult pneumothorax (OR = 37.562, 95% CI 6.754–208.915; *p* ≤ 0.0001). OPTX patients with displaced rib fractures were found to have a 4 times higher odds of a having a chest tube placed (Table 2).

## 4. Discussion

An OPTX is defined as a PTX seen by CT that is not seen on CXR [14–16]. Implicit to this definition is that the OPTX is a small volume of extrapleural air, but there is no defined volume. Carr et al. demonstrated that upto 200 cc extrapleural air could be present without being seen by supine CXR [17]. The definitive management of OPTX remains an ongoing debate. Previous studies have reported that 48%–63.3% of OPTX cases can be managed conservatively without chest tube placement [18, 19]. In our study, the majority of OPTX patients (67.94%) were successfully managed without chest tube placement. The low chest tube insertion rate (32.06%) implies that a selective treatment approach was actively used for these patients, consistent with current trends in thoracic trauma [20].

When managing patients with occult pneumothorax, clinicians need to have a high index of suspicion for progression of the pneumothorax and deterioration of the patient, a concept previously known as delayed pneumothorax [8, 9]. As previous studies have reported, not every patient can be successfully managed with observation. Identifying a pneumothorax on CT scan after not having seen it on chest X-ray changes the management plan in up to 41% of patients [21]. Tube thoracostomy has its own set of risks and is associated with complication rate as high as 22% [3]. The in-hospital complication rate and hospital and intensive care unit length of stays in patients that received a chest tube were significantly higher than those that did not receive a chest tube (Table 1). As is the case in the current study, the patients with an OPTX who did not undergo chest tube placement had a lower rate of complications and a shorter length of stay in the hospital [19, 22].

Although there are studies supportive of observation of most of the blunt trauma patients with an OPTX, the potential for a failure of observation exists, most commonly due to progression of OPTX, respiratory distress, or development of hemothorax [4, 23, 24]. Kirkpatrick et al. reported that one-third of patients with an OPTX who were treated for a week in an ICU eventually required chest tube placement [25]. Chest tube placement is also advised in patients on positive pressure ventilation to prevent development of tension pneumothorax [3, 26]. Indeed, failing to place a chest tube in an OPTX could lead to the serious and potentially fatal complication of tension pneumothorax [27]. It is therefore vital that the clinician make the best choice regarding the modality of treatment for the patient with an OPTX.

According to our data, patients with endotracheal intubation, a moderate-sized pneumothorax, and those with displaced rib fractures were more likely to receive a chest tube as treatment of an OPTX. Similar to findings of our study, Hefny et al. found endotracheal intubation and pneumothorax volumes are associated with chest tube insertion in patients with an OPTX [18]. Past studies with regards to OPTX did not investigate the association of type of rib fractures that patients have, i.e., displaced versus nondisplaced with chest tube placement. Chien et al. identified that the number of displaced rib fractures is strongly associated with pulmonary complications. Additionally, displaced rib fractures also have the potential to penetrate the pleura into the lung parenchyma [28]. Therefore, chest tube placement can be more rigorously considered in OPTX patients with displaced rib fractures. Future studies could also evaluate the degree of rib fracture displacement, to determine if the gradation of displacement affects the success of observation vs. thoracostomy tube placement.

We believe there is value in reviewing the data of the patients whose OPTX progressed. Our extensive review of the literature failed to identify any publications which address the optimal time for the first follow-up CXR in the absence of changes in the patient's clinical picture [3, 4, 22, 29]. Four patients were noted to have progression of the OPTX on their first follow-up CXR, which were performed at 8, 12, 17, and 19 hours. Perhaps more importantly, two patients had no progression of their OPTX seen on their first follow-up CXR at 2 and at 3 hours but were seen to have progression of their OPTX on the second follow-up CXR at 17 and at 26 hours. In total, 6 of 100 patients with OPTX initially selected for period of observation had progression of their PTX to be visible on subsequent CXRs (6%). Kiev previously reported that 4.2% of such patients had progression at a 3-hour follow-up CXR [9]. Based on our very limited number of 6 patients whose OPTX were seen to progress on follow-up CXRs, it appears that the first routine follow CXR should not occur in less than 4–8 hours, in the absence of clinical signs of deterioration. This conclusion is somewhat at odds with the conclusions of Kiev, as 2 of our 6 patients demonstrated progression after 3 hours [9]. But clearly this warrants further investigation with a significantly larger number of patients.

One of the weaknesses of our study is the lack of exact quantification of the size of either the OPTX or lost lung volume. In our radiology reports, pneumothoraces were simply classified as small, moderate, or large. There is no definite system in place for radiologists to uniformly quantify the size of a pneumothorax on CT. Sayar et al. examined the size of pneumothoraces, identifying large pneumothoraces as greater than 50% loss of lung volume, whereas small and moderate were considered <50% loss of lung volume, with small generally defined as <15% loss of lung volume [30]. As size of the pneumothorax documented by radiology was one of the variables that we found to be significantly associated with chest tube placement in OPTX patients, we should therefore aim to have a more uniform measurement of pneumothorax volume. Working with our staff radiologists to objectively measure the size of the pneumothoraces on CT scan rather than calling them small, moderate, or large could aid in the management of these patients. Another limitation of our study was that the location of OPTX was not identified. It is helpful to know the location as it was previously reported that basal and bilateral OPTXs may benefit from chest tube placement, while apical OPTX are more likely to be successfully observed [31].

Our study also suffers from the lack of standardized follow-up imaging after the initial diagnosis of OPTX was made. Future studies could set a temporal parameter for repeat CXRs at a given time period to evaluate for progression or stability of the OPTX.

## 5. Conclusions

Occult pneumothorax is a more common diagnosis now than in the past with the increasing usage of CT scanning in the trauma patient. With this diagnosis comes the question of whether or not to intervene. Our study has identified displaced rib fractures, intubation, and a moderate-sized pneumothorax as significant factors associated with chest tube placement in blunt trauma with an OPTX. The results of this study may assist in the management of OPTX in trauma patients, thereby limiting unnecessary interventions and their associated complications.

## Figures and Tables

**Table 1:** Demographic and clinical characteristics of the study sample.

Variable	Total sample (*n* = 131)	Chest tube (*n* = 42)	No chest tube (*n* = 89)	*p* value
Age	44.48 ± 22.09	48.67 ± 20.46	42.51 ± 22.67	0.1370
Sex				
Female	45 (34.35%)	13 (30.95%)	32 (35.96%)	0.5736
Male	86 (65.65%)	29 (69.05%)	57 (64.04%)	
Number of fractured ribs	4.33 ± 4.18	5.98 ± 4.21	3.55 ± 3.95	0.0017^*∗*^
Patients with rib fractures	97 (74.05%)	38 (90.48%)	59 (66.29%)	0.0032^*∗*^
Rib fracture type				
Displaced	53 (54.64%)	27 (71.05%)	26 (44.07%)	0.0056^*∗*^
Nondisplaced	44 (45.36%)	11 (28.95)	33 (55.93%)	
OPTX size				<0.0001^*∗*^
Small	112 (85.50%)	26 (61.90%)	86 (96.63%)	
Moderate	16 (12.21%)	13 (13.95%)	3 (3.37%)	
Large	2 (1.52%)	2 (4.76%)	0 (0.00%)	
Unknown	1 (0.7%)	1 (2.38%)	0 (0.00%)	
Smoking status				0.0656
Active	26 (19.85%)	8 (19.05%)	18 (20.22%)	
Never	88 (67.18%)	25 (59.52%)	63 (70.79%)	
Quit	7 (5.34%)	2 (4.76%)	5 (5.62%)	
Unknown	10 (7.63%)	7 (16.67%)	3 (3.37%)	
Intubated patients	47 (36.15%)	31 (75.61%)	16 (17.98%)	<0.0001^*∗*^
Mechanism of injury				0.3657
Bicyclist	4 (3.05%)	2 (4.76%)	2 (2.25%)	
Fall	26 (19.85%)	6 (14.28%)	20 (22.47%)	
Motor vehicle crash	62 (47.33%)	19 (45.24%)	43 (30.16%)	
Motor cycle crash	17 (12.98%)	4 (9.52%)	13 (14.61%)	
Pedestrian struck	20 (15.27%)	10 (23.81%)	10 (11.24%)	
Hit by blunt instrument	2 (1.53%)	1 (2.38%)	1 (1.12%)	
Oxygen saturation	97.47 ± 4.0	96.98 ± 5.80	97.71 ± 2.78	0.4458
Number of chest X-rays	6.34 ± 7.26	12.69 ± 9.41	3.34 ± 2.88	<0.0001^*∗*^
Hospital length of stay	9.69 ± 12.86	18.29 ± 17.88	5.63 ± 6.59	<0.0001^*∗*^
ICU length of stay	3.41 ± 5.08	6.64 ± 6.85	1.89 ± 3.00	<0.0001^*∗*^
ISS	20.99 ± 10.95	25.76 ± 14.15	18.74 ± 8.23	0.0042^*∗*^
RTS	7.33 ± 1.16	6.76 ± 1.61	7.59 ± 0.76	0.0031^*∗*^
POS	0.89 ± 0.18	0.80 ± 0.23	0.93 ± 0.14	0.0020^*∗*^
Prehospital RR	17.07 ± 6.03	16.11 ± 6.72	17.64 ± 5.57	0.2365
Prehospital PO_2_	91.35 ± 23.34	87.82 ± 23.72	93.06 ± 23.30	0.4536
Prehospital GCS	12.74 ± 4.02	12.03 ± 4.38	13.16 ± 3.78	0.2277
Mortality	12 (9.16%)	7 (16.67%)	5 (5.62%)	0.0408^*∗*^
Complication rate	31 (23.66%)	15 (35.71%)	3 (3.37%)	0.0003^*∗*^

*Notes.* ^*∗*^ *p* < 0.05; OPTX = occult pneumothorax; ICU = intensive care unit; ISS = Injury Severity Score; RTS = Revised Trauma Score; POS = probability of survival; RR = respiration rate; PO_2_ = partial pressure of oxygen; GCS = Glasgow Coma Scale.

**Table 2:** Multivariable logistic regression analysis of factors associated with chest tube placement.

Variable	Odds ratio	95% CI	*p* value
Displaced rib fracture	4.166	1.455–11.930	0.0078^*∗*^
Intubation	15.581	4.676–51.914	<0.0001^*∗*^
ISS	1.032	0.980–1.088	0.2341
Moderate-sized OPTX (reference small OPTX)	37.562	6.754–208.915	<0.0001^*∗*^

*Notes.* CI = confidence interval; ^*∗*^*p* < 0.05; ISS = Injury Severity Score; OPTX = occult pneumothorax.

## Data Availability

Deidentified data and material can be provided upon request.
